# APSIC guidelines for the prevention of surgical site infections

**DOI:** 10.1186/s13756-019-0638-8

**Published:** 2019-11-12

**Authors:** Moi Lin Ling, Anucha Apisarnthanarak, Azlina Abbas, Keita Morikane, Kil Yeon Lee, Anup Warrier, Koji Yamada

**Affiliations:** 1 Infection Prevention & Epidemiology, Singapore General Hospital, Singapore, 169608 Singapore; 2 Infectious Diseases, Division of Infectious Diseases, Thammasat University Hospital, Khlong Nueng, Thailand; 3 Orthopaedic Surgery, National Orthopaedic Centre of Excellence for Research and Learning (NOCERAL), Faculty of Medicine, University of Malaya, Kuala Lumpor, Malaysia; 4 Division of Clinical Laboratory and Infection Control Yamagata University Hospital, Yamagata, Japan; 5 Surgery, Medical College, Kyung Hee University Center, Gangdong-gu, South Korea; 6 Infectious Diseases and Infection Control, Aster Medcity, Kochi, India; 7 Orthopaedic Surgery, Kanto Rosai Hospital, Kawasaki, Japan

**Keywords:** Surgical site infection, SSI, Prevention, Safe surgery

## Abstract

**Background:**

The Asia Pacific Society of Infection Control (APSIC) launched the APSIC Guidelines for the Prevention of Surgical Site Infections in 2018. This document describes the guidelines and recommendations for the setting prevention of surgical site infections (SSIs). It aims to highlight practical recommendations in a concise format designed to assist healthcare facilities at Asia Pacific region in achieving high standards in preoperative, perioperative and postoperative practices.

**Method:**

The guidelines were developed by an appointed workgroup comprising experts in the Asia Pacific region, following reviews of previously published guidelines and recommendations relevant to each section.

**Results:**

It recommends that healthcare facilities review specific risk factors and develop effective prevention strategies, which would be cost effective at local levels. Gaps identified are best closed using a quality improvement process. Surveillance of SSIs is recommended using accepted international methodology. The timely feedback of the data analysed would help in the monitoring of effective implementation of interventions.

**Conclusions:**

Healthcare facilities should aim for excellence in safe surgery practices. The implementation of evidence-based practices using a quality improvement process helps towards achieving effective and sustainable results.

## Introduction

The incidence of SSI globally varies from 0.9% of cumulative SSI rate in the USA (NHSN 2014), to 2.6% in Italy, 2.8% in Australia (2002–13, VICNISS), 2.1% in Republic of Korea (2010–11) to 6.1% in Low Middle Income Countries (LMIC) (WHO, 1995–2015) and 7.8% in South East Asia (SEA) & Singapore (pooled incidence from 2000 to 2012) [personal communication]. What definitely stands apart is the very high incidence rates in LMIC and SEA compared to the USA, Europe and Australia. This highlights the need for the SEAsian countries to look at the specific risk factors and develop effective prevention strategies, which would be cost effective at local levels. A summary of common general risk factors may be seen in Table 1.

**Table 1:** Risk factors for SSIs

Preoperative risk factors 1. Unmodifiable a. Increasing age until age 65 years b. Recent radiotherapy and history of skin or soft tissue infection 2. Modifiable a. Uncontrolled diabetes b. Obesity, malnutrition c. Current smoking d. Immunosuppression e. Preoperative albumin < 3.5 mg/dL f. Total bilirubin > 1.0 mg/d g. Preoperative hospital stay of at least 2 days	
Perioperative risk factors 1. Procedure-related a. Emergency and more complex surgery, b. Higher wound classification c. Open surgery. 2. Facility risk factors a. Inadequate ventilation, b. Increased operation theatre traffic c. Inappropriate/inadequate sterilization of instruments/equipment. 3. Patient preparation-related a. A pre-existing infection b. Inadequate antiseptic skin preparation c. Preoperative hair removal d. Wrong antibiotic choice, administration, and/or duration 4. Intraoperative risk factors a. Long operating time b. Blood transfusion c. Asepsis and surgical technique d. Hand/forearm antisepsis and gloving techniques e. Hypoxia f. Hypothermia g. Poor glycaemic control.	
Postoperative risk factors 1. Hyperglycaemia and diabetes 2. Postoperative wound care 3. Transfusion	

The full APSIC Guidelines for the Prevention of Surgical Site Infections is available at https://apsic-apac.org as reference to guide practice. It is developed to assist countries to implement best practices to prevent SSIs esp. in low resource setting.

### Review workgroup composition

APSIC convened experts in Infection Prevention and surgical discipline from Asia Pacific region to develop the APSIC Guidelines for the Prevention of Surgical Site Infections. The members of this workgroup are the authors of this paper.

### Literature review and analysis

For the development of this APSIC guideline, the workgroup reviewed previously published guidelines (e.g. WHO, CDC, Cochrane, etc.) and recommendations relevant to each section and performed computerized literature searches using PubMed. Examples of key search terms used include SSI, prevention and the various topics reviewed.

### Process

The workgroup met on 2 occasions as well as discussed via email correspondences to complete the development of the guideline. Criteria for grading the strength of recommendation and quality of evidence are described in Table 2. The draft was then submitted to two external reviewers, APSIC Executive Committee and national Infection Control societies in Asia Pacific. Comments obtained were then reviewed by the workgroup for necessary edits, following which the final copy was circulated for approval and endorsement by the APSIC Executive Committee and national societies from the Asia Pacific region.

**Table 2:**
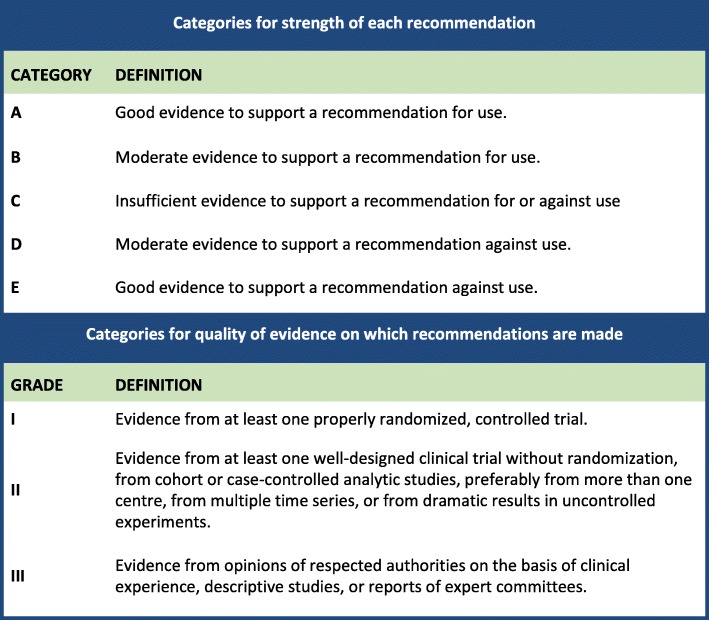
Categories for strength of each recommendation

### Recommendations for surveillance of surgical site infections (SSIs)

Surveillance is a systematic methodology which includes monitoring of a specific event, collection and analysis of necessary data associated with the event, and the timely feedback to clinical staff who can implement evidence-based strategies to improve outcomes by decreasing the incidence of the event. Surveillance of SSI with feedback of appropriate data to surgeons and other healthcare workers involved in the care of those undergoing operative procedures has been shown to be an important component of strategies to reduce the risk of SSIs [1, 2]. A successful surveillance program includes the use of standardized SSI definitions and surveillance methods, stratification of SSI rates according to risk factors associated with SSI development, and timely feedback of data [3].

1. *Perform surveillance of SSIs using accepted international methodology. (IIB)*

### Recommendations for pre-operative preventive measures

#### Pre-operative bath

It is generally accepted that preoperative bathing with soap (antimicrobial or non-antimicrobial) is beneficial prior to surgery, despite the lack of study comparing preoperative bath versus no-preoperative bath on the occurrence of SSIs.

Although recommendations on preoperative bathing in relation to time of administration and the most effective protocol for perioperative bath remains an unresolved issue, it is advisable to take at least 2 baths pre-operatively [4]. Countries with high incidence of multidrug resistant organisms may want to consider the use of an antiseptic instead of plain soap as a preoperative bath. In some Asian countries where allergy to chlorhexidine (CHG) is common or CHG is not available, alternative agents such as octenidine may be used.

1. It is necessary for patients who will undergo surgery to have at least 1 preoperative bath with soap (antimicrobial or non-antimicrobial). (IIB)

#### Mechanical bowel preparation (MBP) and oral antibiotics for elective colorectal surgery in adults

Oral antibiotics have been used to decrease the luminal bacterial load since the 1930s, but it does not decrease SSI. Similarly, oral or intravenous antibiotics alone showed suboptimal effects. A 2014 Cochrane review also recommended that antibiotics should be administered both orally with mechanical bowel preparation and intravenously in 1 h before surgery to reduce SSIs [5].

1. Combination mechanical bowel preparation and oral antibiotic preparation are recommended for all elective colorectal surgery in adults. (IA)

#### Hair removal

There are several methods to remove hair at the surgical site preoperatively. Hair removal by shaving and the night before an operation is associated with and increased risk of SSI. Shaving and/or clipping can cause microscopic cuts in the skin that later serve as foci for bacterial multiplication [6, 7].

*1. Hair removal should be avoided unless hair interferes with the operative procedure. (IIIB).*

*2. If hair removal is necessary, a razor should be avoided and an electric clipper should be used. (IA).*

*3. No recommendation regarding the timing of hair removal by clipper is made. (IIIC).*

#### Methicillin-resistant *Staphylococcus aureus* (MRSA) screening and decolonization

It is well recognized that MRSA colonization is associated with worse outcomes and a higher risk for both MRSA SSI and overall SSl. The use of MRSA bundle comprising of screening, decolonization, contact precautions, and vancomycin-containing antibiotic prophylaxis was associated with decreased rates of SSI where there was high compliance with the bundle strategies [8–10].

1. *Hospitals should evaluate their SSI, Staphylococcus aureus (S. aureus) and MRSA rates, and mupirocin resistant rate, if available, to determine whether implementation of a screening program is appropriate. (IIB)*
2. Patients undergoing cardiothoracic and orthopedic surgery with known nasal carriage of *S. aureus* should receive perioperative intranasal application of mupirocin 2% ointment with or without a combination of CHG body wash. (IA)

#### Surgical hand/forearm preparation

The objective of cleaning hands and forearms prior to surgery is to reduce the bioburden of bacteria on the skin of the surgical team. The second objective is to inhibit the growth of bacteria. Hands and forearms should undergo a surgical scrub with a surgical antiseptic. When using alcohol-based hand rub (ABHR) solutions containing 60–80% alcohol is recommended. Water quality may be compromised with the use of tap aerators where these are known to be easily colonized with non-fermentative Gram-negative bacteria e.g. *Pseudomonas aeruginosa, Acinetobacter baumannii*, etc. Hence, where there are issues with the quality of water used in rinsing hands after hand scrubbing, hand rubbing with ABHR agent is a suitable alternative [11–13].

1. *Surgical hand preparation is to be performed either by scrubbing with a suitable antiseptic soap and water or a suitable ABHR before donning sterile gown and gloves. (IA)*
2. *ABHR used in surgical hand preparation should comply with EN 12791 or ASTM E-1115 standards. (IIIA)*
3. *Where the quality of water used is not assured, surgical hand rub with ABHR is recommended. (IIIB)*

#### Skin antiseptic

Current evidence suggested that alcohol-based preparations are more effective in reducing SSI than aqueous preparations, and should be used, unless contraindicated [14, 15]. Alcohol has a rapid bactericidal effect, albeit with the lack of persistent antibacterial effect. The benefit of iodine or chlorhexidine and alcohol solutions is prolonged bactericidal activity [16].

1. Alcohol based skin antiseptic preparations should be used, unless contraindicated. (IA)

#### Surgical prophylaxis

Current guidelines suggest the use of narrow spectrum antibiotics, such as cefazolin for most surgical procedures, or cefoxitin for abdominal surgery, as surgical antimicrobial prophylaxis. In situations where the incidence of MRSA-associated SSI is high or in case/s of penicillin allergy, vancomycin or fluoroquinolone can be used as an alternative. Current evidence supports the administration of an antimicrobial for surgical prophylaxis within 1 h before incision or before inflation of a tourniquet in orthopaedic procedures, or within 2 h for vancomycin or fluoroquinolones, because of their recommended infusion times [17, 18]. In most cases, it is recommended that a single dose of surgical antimicrobial prophylaxis is adequate.

1. *Administration of prophylaxis antimicrobials should only be performed when indicated. (IA)*
2. *Prophylactic antimicrobials should be administered within 1 h before incision for all antimicrobials except vancomycin and fluoroquinolones where it should be administered within 2 h. (IA)*
3. *Re-dosing should be considered to maintain adequate tissue levels based on agent half-life. (IA)*
4. *A single dose of antimicrobial prophylactic is adequate for most surgical procedures. (IA)*

#### Nutrition

Changes in host immunity may increase a patient’s susceptibility to SSIs and malnutrition may contribute to poor surgical outcomes, including delayed recovery, morbidity and mortality, prolonged hospital stay, increased health care costs and readmission. Meta-analysis and randomized controlled studies do not consistently show either benefit or harm when comparing standard versus enhanced nutritional support in reducing the risk of SSIs [19–22]. Underweight patients undergoing major surgical procedures, especially oncology and cardiovascular operations, however, may benefit from enhanced nutritional support.

1. *Underweight patients undergoing major surgical procedures, especially oncology and cardiovascular operations, may benefit from the administration of oral or enteral multiple nutrient-enhanced nutritional formulas for the purpose of preventing SSI. (IIIC)*

#### Glycemic control

One of the commonest surgical complications in patients with pre-existing diabetes mellitus and hyperglycemia is infection, with superficial surgical site infections (SSIs), deep wound infections, surgical space abscesses, urinary tract infections and pneumonia accounting for a large percentage of infectious complications. The American Diabetes Association defines poorly controlled diabetes as having a target HbA1c level of ≥8% [23]. Using this threshold, studies have shown a higher occurrence of postoperative wound infections in cardiac and orthopaedic patients who had HbA1C levels of ≥8% [24, 25]. To optimize the care of the patient with diabetes and reduce the risk of complications, a team-oriented approach to treatment is highly recommended [26–28].

1. *Preoperative HbA1C levels should be less than 8%. (IIIC)*

### Recommendations for intra-operative preventive measures

#### Normothermia

Exposure of large surfaces of skin to cold temperatures in the operating room can cause hypothermia. Hypothermia results in patients waking with chills and shivering, and also raises the risk for other complications such as SSI [29–32]. To avoid these complications, warming systems to transfer heat to a patient’s body are used. Several different methods are available, including a forced-air warming system, water bed system, and passive warming system such as blankets.

1. *Maintain perioperative normothermia by using active warming devices. (IB)*

#### Normovolemia

Hypovolemia and reduced cardiac output theoretically trigger musculocutaneous and splanchnic vasoconstriction, causing hypoperfusion and tissue hypoxia. Hemodynamic goal-directed therapy, a treatment based on the titration of fluid and inotropic drugs infused to physiologic flow-related end points, is shown to significantly reduce SSIs by 42% in a systematic review [33, 34].

1. *Hemodynamic goal-directed therapy is recommended to reduce surgical site infection. (IA)*

#### Irrigation

Wound irrigation is considered to be one of the most useful SSI prevention methods by many surgeons. We agree with WHO and NICE that there is inadequate evidence to comment on this and concur with WHO that antibiotic irrigation for SSI prevention should be avoided [35, 36].

*1. There is insufficient evidence to recommend for or against saline of incisional wounds before closure for the purpose of preventing SSI. (IIC).*

*2. Avoid using antimicrobial agents to irrigate the incisional wounds before closure to reduce the risk of SSI. (IA).*

#### Antimicrobial impregnated sutures

The latest meta-analysis by Leaper et al., focusing on the cost savings from SSI prevented with addition of antimicrobial sutures as a preventive measure (used in all classes of surgeries), suggests benefits [37].

1. *Where there are high SSI rates in clean surgeries, in spite of basic preventive measures, individual centers may consider the use of antimicrobial impregnated sutures. (IIB)*

#### Drapes

In various guidelines, it is generally accepted not to recommend non-iodine- impregnated adhesive incise drapes, since it is associated with SSI risk. However, in several observational studies especially in clean surgeries, marked SSI reduction reported with the proper use of iodine-impregnated drapes [38–42]. Considering the promising effect of controlling skin recolonization, and the fact that bacterial wound contamination may be directly linked to SSI, we believe that the use of iodine- impregnated adhesive incise drapes may be beneficial. Based on the above evidence, we do recommend their use when necessary, especially in orthopedic and cardiac surgeries.

*1. When using adhesive incise drapes, do not use non-iodophor-impregnated drapes for surgery as they may increase the risk of surgical site infection. (IE).*

*2. In orthopedic and cardiac surgical procedures where adhesive incise drapes are used, consider using an iodophor-impregnated incise drape, unless the patient has an iodine allergy or other contraindication. (IIB).*

#### Wound protectors

In the WHO Global Guidelines for the prevention of SSI, the expert panel concluded that the use of a wound-protector device (single-ring or double-ring) was associated with a significantly lower risk of SSI than with conventional wound protection (OR 0·42; 95% CI 0·28–0·62). Unfortunately, the quality of evidence was too low to justify a recommendation to routinely use wound protectors. In resource limited countries, these single use devices may be financially prohibitive [10, 43–45].

1. *Careful evaluation of wound protectors needs to be done before introducing the use of wound protectors as a routine measure to reduce SSI. (IIIC)*

#### Vancomycin powder

We concur with the Centers for Disease Control and Prevention (CDC), where in its latest guidelines strongly recommended not to apply antimicrobial agents (i.e. ointments, solutions, or powders) to the surgical incision for the prevention of SSI [46]. Though many studies provide supportive results, several serious concerns exist regarding their study designs, including the randomized controlled trials (RCTs) [47, 48]. With global concern on antimicrobial resistance, we will need to discourage the unnecessary resistance pressure associated with use, leading to vancomycin resistant *Staphylococcus aureus* (VRSA). Hence, vancomycin powder is not recommended for the purpose of preventing SSIs at this point, including spinal surgery.

1. *Do not apply vancomycin powder into the surgical site for prevention of surgical site infection, including spine surgery. (IC)*

#### Laminar air flow

Heterogeneity is seen with data published, and lack of standardization is noted in the surveillance methods and registers used regarding the use of laminar air flow and its association with SSI. In the latest meta-analysis from the WHO, with some additional studies, the risk for deep SSI in association with laminar air flow showed no significant difference compared with a conventional air flow system, with OR: 1.08(95% CI 0.77–1.52, *p* = 0.65) for knee arthroplasty, OR: 1.29 (95% CI 0.98–1.71, *p* = 0.07) for hip arthroplasty, and OR: 0.75(95% CI 0.43–1.33, *p* = 0.33) for abdominal and open vascular surgeries. Therefore, WHO has suggested laminar air flow is not required to reduce the risk of SSI for patients undergoing total arthroplasty surgery, and laminar air flow is not required in new operating rooms. Due to the high expense, laminar air flow is not considered necessary for installation in new operating rooms, unless supportive sufficient clinical evidence has been provided.

1. *Installation of laminar airflow is not required in new or renovated operating rooms to prevent SSIs. (IIC)*

### Recommendations for post-operative wound management

Unfortunately**,** there is a lack of high-quality studies comparing various strategies of post - operative wound management and this certainly an area for further focused research. Aseptic technique should be used when undertaking wound dressings and wound management. Choice of dressing will depend on patient and wound needs, i.e. exudate level, wound depth, need for conformability, antimicrobial efficacy, odor control, ease of removal, safety and patient comfort [49–52].

1. *Primary vacuum dressings or Negative Pressure Wound Therapy (*i.e. *for clean-contaminated and contaminated surgeries) and silver-based dressings have mixed results and individualized decisions on their use are suggested. Routine use for prevention of SSI is not recommended. (IIC)*

## Conclusion

We recommend hospitals in the Asia Pacific region that have high surgical site infection rates to consider reviewing their practices in accordance with the Guidelines for the Prevention of Surgical Site Infections to identify areas for improvement. We have chosen not to identify variables for a bundle. Instead, a gap analysis is recommended comparing current practices with the various recommendations in the guidelines. This should then be followed by a process improvement plan using the approach described in the APSIC Guide for Prevention of Central Line Associated Bloodstream Infections (CLABSI) to close the gaps identified [53].

Further studies are needed to demonstrate cost-effectiveness of prevention of SSIs using the process improvement approach, especially in a resource constrained setting.

## Data Availability

The authors reviewed previously published guidelines and recommendations relevant to each section and performed computerized literature searches using PubMed.

## Abbreviations

ABHR: Alcohol-based hand rub
APSIC: Asia Pacific Society of Infection Control
ASTM: American Society for Testing and Materials
CDC: Center for Disease Control and Prevention
CHG: Chlorhexidine
CLABSI: Central line associated blood stream infection
EN: European Norms
HbA1C: Hemoglobin A1C
LMIC: Low Middle Income Countries
MBP: Mechanical bowel preparation
MRSA: Methicillin resistant *Staphylococcus aureus*
NHSN: National Healthcare Safety Network
NICE: National Institute for Health and Care Excellence
RCT: Randomized controlled trials
SEA: South East Asia
SSI: Surgical site infection
USA: United States of America
VRSA: Vancomycin resistant *Staphylococcus aureus*
WHO: World Health Organization
